# Dual-Mode Photonic Synapse Based on a Lead-Free 2D Ruddlesden–Popper Perovskite for Neuromorphic Vision

**DOI:** 10.1021/acsami.5c10557

**Published:** 2025-08-16

**Authors:** Cheng-Yueh Chen, Hao-Cheng Lin, Pei-En Jan, Hung-Ming Chen, Yung-Tang Chuang, Chia-Feng Li, Yu-Ching Huang, Hao-Wu Lin

**Affiliations:** † Department of Materials Science and Engineering, 34881 National Tsing Hua University, Hsinchu 30013, Taiwan; ‡ Research Center for Critical Issues, Acdamia Sinica, Tainan 711, Taiwan; § Department of Materials Engineering, 56082 Ming Chi University of Technology, New Taipei City 24301, Taiwan

**Keywords:** Ruddlesden−Popper perovskite, lead-free, photodetector, photosynapse, dual-mode

## Abstract

Inspired by the human visual system, photonic synapses with photonic sensing and data memorization offer a promising alternative to traditional von Neumann architectures for neuromorphic computing. This study introduces a multifunctional artificial photonic synapse based on solution-processed PEA_2_SnI_4_ 2D Ruddlesden–Popper perovskite. By modulation of the applied bias voltage, the PEA_2_SnI_4_ device can switch between two distinct optoelectronic modes. In the absence of bias, the device operates in the photodetector mode, demonstrating a responsivity of 42.4 mA W^–1^. The low dark current of the device allows for a high detectivity of 3.6 × 10^14^ Jones and a broad linear dynamic range of 140 dB. Under reverse bias, the device transitions into a synaptic mode, enabling the observation of several synaptic behaviors, including paired-pulse facilitation, long-term potentiation, spike-frequency-dependent plasticity, and spike-number-dependent plasticity. The synaptic behavior is attributed to band alignment and carrier accumulation in the interfacial layer. Moreover, the synaptic performance of the PEA_2_SnI_4_ device is further illustrated through simulations of image contrast enhancement and edge detection. This work reveals the potential of PEA_2_SnI_4_-based photonic synapses for next-generation neuromorphic vision systems, offering an energy-efficient and highly adaptable approach to optoelectronic computing applications.

## Graphical abstract

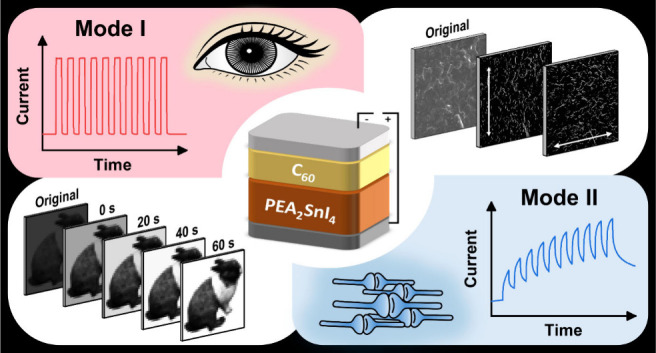

## Introduction

In the human visual system, the retina serves the role of receiving light signals carrying environmental information, and the signals are sent to neurons for further processing. The neurons are capable of executing multiple complex tasks with remarkable parallelism, low energy consumption, fault tolerance, and robustness.^1,2^ Inspired by the human brain, neuromorphic computing shows great potential for time and energy efficient massive data computing compared to traditional von Neumann architecture-based computing system.^3–6^ The ability of biological synapses to both compute and store information simultaneously enables the human brain to process vast amounts of information in parallel with low energy consumption.^7–9^ In recent published reports, all-electronic artificial synapses have been widely explored.^10–13^ However, more than 70% of the information for humans to respond to the external environment is derived visually.^14^ Thus, the importance of photonic synapses has recently drawn increasing attention from researchers recently. The photonic sensing and neuromorphic computing behavior enable photonic synapses to be applied in fields such as direct pattern recognition, neuromorphic vision sensing, and collision detection.^15–18^

In previous reports, artificial synapses have been successfully proposed using various materials such as metal oxides,^19,20^ organic materials,^21,22^ and low-dimensional materials.^23,24^ Among these materials, halide perovskites offer great prospects for optoelectronic applications, owing to their outstanding properties. The characteristics of long charge carrier lifetime, small exciton binding energy, and tunable bandgap enable halide perovskites to be promising materials for optoelectronic applications such as solar cells, light-emitting diodes,^25^ and photodetectors.^26–28^ Notably, Sn-based perovskites with low toxicity, long diffusion length, large light absorption coefficients, and high carrier mobility are considered favorable candidates to replace lead-based perovskite.^29–32^ However, the small formation energy of Sn vacancy defects resulting from the easy oxidation of Sn^2+^ to Sn^4+^ leads to significant harm to the stability and performance of devices.^33,34^ Compared to 3D Sn-based perovskites, 2D Ruddlesden–Popper Sn-based perovskites have lower self-doping concentrations and less ion migration benefiting from the quantum confinement effect, which improves device efficiency and stability.^35,36^

In this study, a multifunctional artificial photonic synapse with a solution-processed PEA_2_SnI_4_ 2D Ruddlesden–Popper perovskite is demonstrated. By adjustment of the applied bias voltage, the PEA_2_SnI_4_ device can switch between two distinct optoelectronic modes. When operating in the photodetector mode, the device achieves a maximum responsivity (*R*) of 42.4 mA W^–1^. The device also exhibits a low noise current, a high detectivity (D*), and a large linear dynamic range (LDR). Under reverse bias, the PEA_2_SnI_4_ device switches to the synaptic mode. Several representative synaptic behaviors such as paired-pulse facilitation (PPF), long-term potentiation (LTP), spike-frequency-dependent plasticity (SFDP), and spike-number-dependent plasticity (SNDP) are achieved due to the carrier accumulated between interfacial layer. Finally, to demonstrate the synaptic behavior of the PEA_2_SnI_4_ device, simulations of image contrast enhancement and image edge detection are presented.

## Results and Discussion

A device structure of ITO/PEDOT:PSS/PEA_2_SnI_4_/C_60_/LiF/Al was used, as shown in Figure 1(a). The lead-free 2D perovskite PEA_2_SnI_4_ was selected as the light absorption layer, while PEDOT:PSS and C_60_ were chosen as the hole transporting layer (HTL) and electron transporting layer (ETL), respectively. The perovskite film was fabricated by spin-coating a mixed solution of PEAI and SnI_2_ (molar ratio of 2:1) in DMF and DMSO (volume ratio of 4:1). A small amount of Sn powder was added to the solution to prevent oxidation of Sn^2+^. After the spin-coating process, the films were annealed at different temperatures ranging from 50 to 150 °C. The UV–vis absorption spectra of the PEA_2_SnI_4_ films with various annealing temperatures are shown in Figure S1. The film annealed at 100 °C shows the highest absorbance, indicating a better formation of the perovskite film. Figure 1(b) shows scanning electron microscopy (SEM) images of the perovskite thin film. From the top view SEM image, the film exhibits complete coverage and a pinhole-free morphology. The cross-sectional SEM image reveals a dense film with a thickness of about 400 nm, without a column-like grain structure. To further estimate the surface roughness of the perovskite film, AFM measurement was conducted, and the result is shown in Figure S2. The root-mean-square roughness of the thin film is calculated to be 18.7 nm. X-ray diffraction (XRD) was also performed to examine the crystallographic properties of the perovskite film. As shown in Figure 1(c), the perovskite film displays the characteristic (00*l*) structure (*l* = 2, 4, 6,···) of 2D perovskite, which is consistent with previous reports.^37,38^ The sharp peaks in the XRD pattern confirm the high crystallinity of the PEA_2_SnI_4_ films. The grazing incidence wide-angle X-ray scattering (GIWAXS) patterns for the PEA_2_SnI_4_ films with annealing temperatures of 50, 100, and 150 °C were further conducted. As shown in Figure 1(d–f), all films present peaks along the q_*z*_ direction, indicating that the orientation of the 2D perovskite is parallel to the substrate. The integrated intensity along the q_*z*_ direction is presented in Figure S3. The film annealed at 100 °C displays a higher peak intensity, indicating a stronger preferred orientation and improved crystallinity, which is consistent with the absorption results.

**Figure 1:**
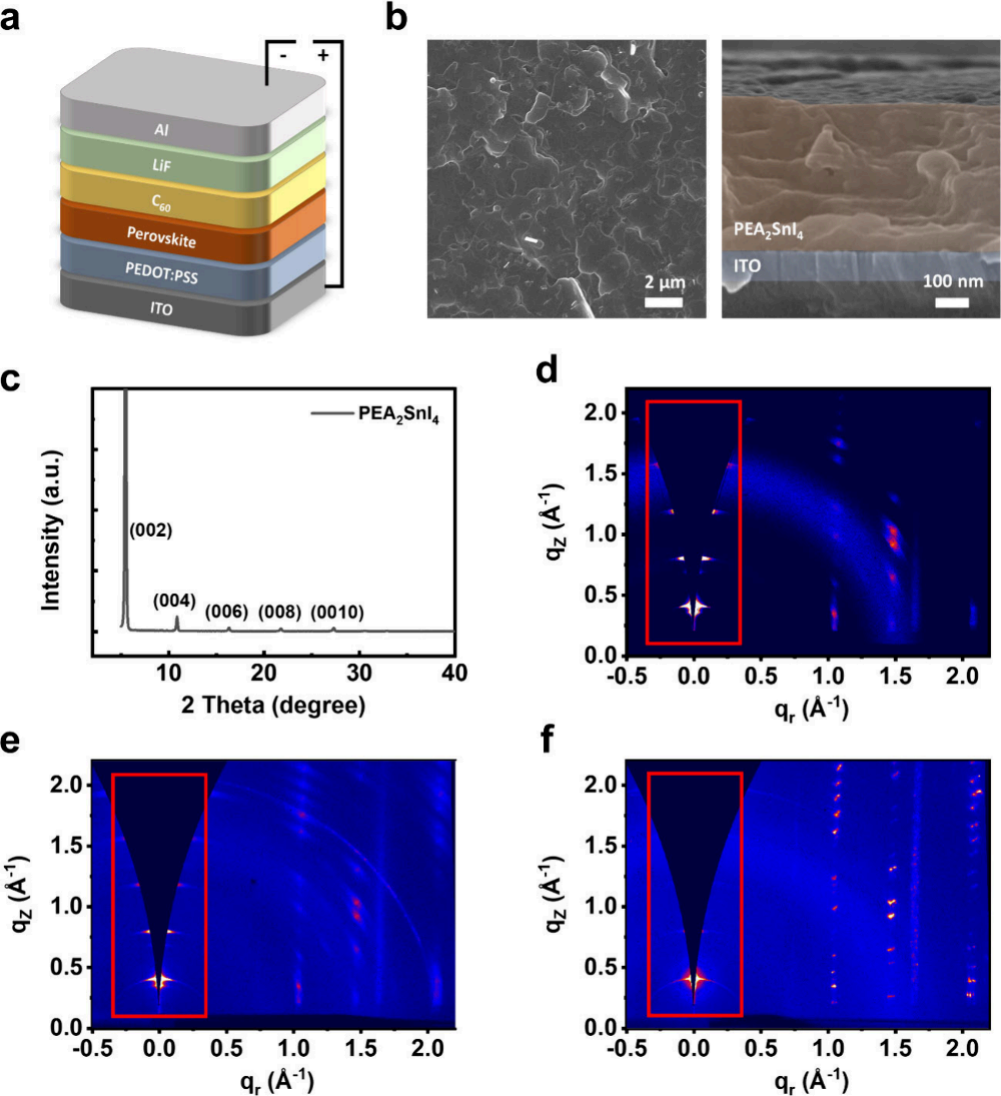
(a) Schematic image of the device structure. (b) Top-view and cross-sectional SEM images of PEA_2_SnI_4_ thin film. (c) XRD patterns of PEA_2_SnI_4_ thin film. GIWAXS patterns of PEA_2_SnI_4_ thin film with annealing temperature of (d) 50, (e) 100, and (f) 150 °C.

Figure 2(a) shows the dark and photocurrent of the device under bias voltage ranging from −1 to +1 V. The device exhibits a low dark current density of 4.06 × 10^–10^ A cm^–2^ at zero bias. The photocurrent densities were measured under air-mass (AM) 1.5G illumination, and the device maintains a photocurrent density of 3.04 × 10^–4^ A cm^–2^ at zero bias. Under AM 1.5G illumination, the device shows an on/off ratio of 7.5 × 10^5^. Figure 2(b) presents the EQE under zero bias, and the maximum EQE value of the device is 8.2%. This maximum EQE value corresponds to a maximum responsivity of 42.4 mA W^–1^, which can be obtained using the following equation:

1 $$R=\left(I_{\mathrm{ph}}-I_{d}\right)/P_{in}$$

where *I*_ph_ is the photocurrent, *I*_d_ is the dark current, and *P*_in_ is the incident light intensity. To estimate the detection limit of the PEA_2_SnI_4_ device, the noise current was obtained by performing a fast Fourier transform (FFT) of the dark current. The results of the instrument and the device are shown in Figure S4, and the noise current of the device is calculated to be 5.47 × 10^–15^ A Hz^–0.5^. With the noise current, the specific detectivity of the device can be calculated by the following equation:

2 $$D^{*}=\frac{\sqrt{AB}}{i_{n}/R}$$

where *A* is the device’s active area, *B* is the bandwidth, and *i*_n_ is the noise current. The maximum D* value is calculated as 3.6 × 10^14^ Jones. Furthermore, the temporal photoresponse of the device was measured under zero bias. As shown in Figure 2(c), 625 nm light pulses with different intensities ranging from 1 to 10 μW cm^–2^ were used. The pulse width and interval between the two light pulses were both set to 1 s. The device exhibits stable switching behavior between the on and off states. The EQE value calculated from the currents of the temporal response is also consistent with the measured EQE. The linear dynamic range represents the range of illuminating light intensity for the constant responsivity of a photodetector. As shown in Figure 2(d), the photocurrent increases linearly with light intensity within the range from 10 nW cm^–2^ to 100 mW cm^–2^ under zero bias. Therefore, the 140 dB LDR of the device has been obtained. The linear fitting of the experimental result shows a mean square deviation (*R* ^2^) of 0.99, indicating the responsivity of the device remains consistent over a wide range of light intensity. Transient photocurrent (TPC) measurement was used to determine the response speed of the device. As shown in Figure 2(e), the rise time of 3.73 μs and the fall time of 10.12 μs were defined as the times when the current changes from 10% to 90% and 90% to 10%, respectively. The Fourier transform of the TPC curve is displayed in Figure 2(f), and the −3 dB bandwidth of the device is estimated to be 4.26 × 10^4^ Hz.

**Figure 2:**
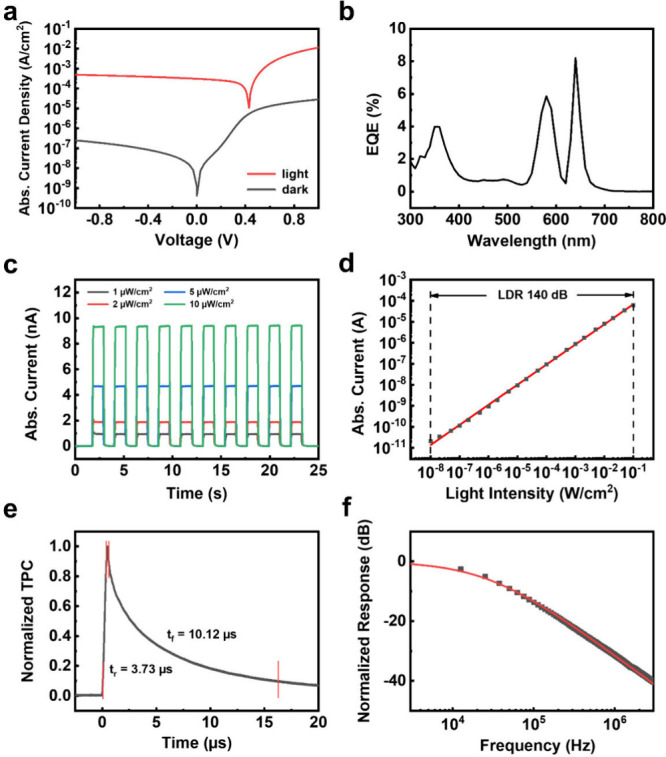
(a) *I*–*V* characteristics of the device under a 1 Sun illumination (red line) and dark (black line) environment. Abs.: absolute. (b) EQE values of the device. (c) Temporal response of the device measured under zero bias. A 625 nm LED light source was used with light intensity vary from 1 to 10 μW cm^–2^, and the on-and-off interval is controlled at 1 s. (d) LDR of the device under various light intensities. The red line represents a linear fit. (e) Transient photocurrent curve of the device measured using a 337 nm pulsed laser as the light source. (f) Frequency response of the PEA_2_SnI_4_ device obtained by performing a fast Fourier transform of the transient photocurrent data.

With the incorporation of reverse bias, the device can operate in synaptic mode. A 625 nm light emitting diode (LED) was used to stimulate the device, and the synaptic behavior of the device is presented in Figure 3(a–f). In biological systems, PPF is a crucial form of short-term synaptic plasticity.^39,40^ When two presynaptic stimuli are fired in quick succession, the second postsynaptic current (PSC) becomes larger than the first one. This mechanism is essential for processing temporal information in sensory modalities, such as auditory and visual signals. Figure 3(a) shows the PPF behavior of the PEA_2_SnI_4_ device under −3 V. The PPF index is defined as the ratio of the second PSC peak (A_2_) to the first PSC peak (A_1_). As the device receives two stimuli, the second photoresponse is amplified, causing a larger PSC compared to the first one. After the stimulus, the PSC does not drop to the initial state, indicating the long-term potentiation behavior of the device. As shown in Figure 3(b), the synaptic behavior of the device is enhanced with the increase in bias voltage. The complete photocurrent of the PPF behavior is shown in Figure S6. As the applied voltage increased from −1 to −3 V, the postsynaptic current of the device increases significantly from a level near the initial state to a much higher value, indicating a transition from short-term memory (STM) to long-term memory (LTM) behavior.^41,42^ The PPF index reaches a maximum value of 126% with the bias voltage of −3 V. To further identify the synaptic behavior, the change of PPF index with different time intervals between two light stimuli was investigated, and the results are shown in Figure 3(c). Unlike other synaptic devices,^43,44^ the PPF index of our device increases as the interval increases from 1 to 10 s, showing an inverse PPF behavior.^45^ This unique behavior originates from the continuous increase in the current under reverse bias. As shown in Figure S7, a slight rise in current is observed when the device operates under reverse bias. As the interval between light pulses increases, the total measurement time also becomes longer, leading to a prolonged duration of reverse-bias operation. This extended operation allows for continuous electron accumulation, resulting in a higher final peak current and the emergence of an inverse PPF behavior. Figure 3(d) presents the SFDP behavior of the device. The SFDP indexes are calculated as the ratio between the first and the 10th PSC peak values at different frequencies (0.1, 0.2, 0.5, 1, and 2 Hz; duty cycle: 50%). The SFDP index at 0.1 Hz was about 151.1% and then gradually decreased to 118.3% at 2 Hz. This SFDP behavior indicated that the synapse is less sensitive to fast and intensive stimuli. Meaningful or infrequent patterns at low frequency are more favorable for the synapse to learn from. The SNDP was measured with different pulse numbers (100, 50, 20, 10), and the results are shown in Figure 3(e). The PSC of the device as well exhibits a transition from STM to LTM with the increasing number of light pulse. The photoresponses with different light pulse intensities were measured and are presented in Figure S8. With an increasing light pulse intensity, the PSC of the device decays to a higher level. Similar to the observation with increasing pulse number and pulse interval, this indicates an STM to LTM transition. For synaptic devices, additional waiting time or inhibitory stimuli are needed to return to their initial state, which can slow down the operation speed or require additional energy consumption. As presented in Figure 3(f), the PSC of PEA_2_SnI_4_ device instantly drops to the initial state when the voltage falls to 0 V, giving the device the ability to operate at a higher speed.

**Figure 3:**
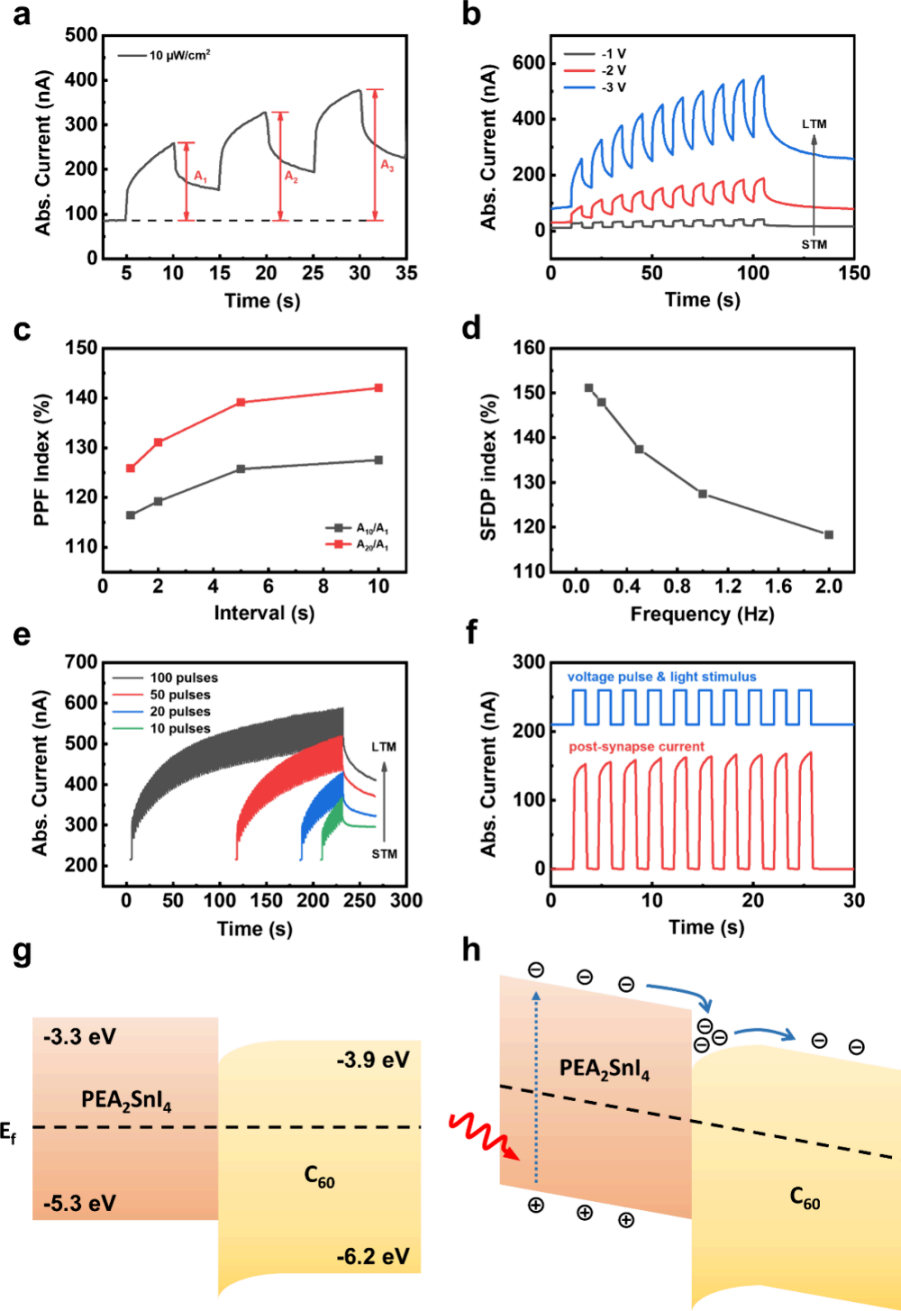
(a) Photoresponse of the device measured under reverse bias of −3 V. A_1_, A_2_, and A_3_ represent the amplitudes of PSC triggered by the first, second, and third light stimulus. (b) Photoresponse of the device under 10 consecutive pulses with various reverse biases from −1 to −3 V. The width and interval of the optical pulse are 5 s. (c) PPF index plotted as a function of the light stimulus interval varied from 1 to 10 s. The width of the optical pulse is 1 s. (d) SFDP index plotted as a function of frequency ranging from 0.1 to 2 Hz. (e) PSC of the device stimulated by different numbers of consecutive light pulses ranging from 10 to 100. The width and interval of the optical pulse are 1 s. (f) Photoresponse of the device under synchronized bias votage and light pulses. PSC of the device drop instantly while the bias voltage and light stimulation ends. (g) Energy band alignment of the device under zero bias and dark environment. (h) Energy band alignment of the device under reverse bias and light illumination.

The synaptic behaviors of the PEA_2_SnI_4_ device are suspected to be attributed to the band alignment between the PEA_2_SnI_4_ and C_60_ layer. To further validate this assumption, devices with different ETLs were fabricated, and the photoresponses under different bias voltages are shown in Figure S9. Under zero bias, all devices demonstrate a photoresponse to 625 nm light pulses. However, the devices with only PO-T2T and BCP show no synaptic behavior under reverse bias. When a C_60_ layer inserts between PEA_2_SnI_4_ and PO-T2T or BCP, the photocurrent increases with the number of pulses. The photoresponses of the devices with C_60_/PO-T2T and C_60_/BCP under reverse bias confirm that the synaptic behavior originates from the interface between the PEA_2_SnI_4_ and C_60_ layers. Figure 3(g) presents the band alignment between PEA_2_SnI_4_ and C_60_ without illumination and bias voltage. The energy levels were determined using ultraviolet photoelectron spectroscopy (UPS), and the results are shown in Figure S10. When the device operates in synaptic mode, the applied reverse bias induces band bending, as illustrated in Figure 3(h), leading to electron accumulation at trap states at the interface. The band bending and the presence of trapped electrons reduce the energy barrier for carrier transport, thereby increasing the dark current and giving rise to inverse paired-pulse facilitation (PPF) behavior. When the device is illuminated by the incident light, the excess charges further enhance the accumulation and reduction of the energy barrier, resulting in a larger photocurrent. To further validate this phenomenon, KPFM measurements were conducted with a pristine perovskite thin film and a perovskite/C_60_ (1 nm) thin film. As shown in Figure S11, the difference in the contact potential difference (CPD) measured under dark and illuminated conditions for the perovskite/C_60_ thin film is larger than that measured for the pristine perovskite thin film. It is worth noting that the KPFM scan was performed from bottom to top. During the measurement, electrons in the perovskite/C_60_ thin film continuously accumulated at the interface, leading to a progressively increasing CPD along the scan direction. This phenomenon is clearly observed in Figure S11­(c), where the CPD under illumination gradually increases along the dashed line from bottom to top. The slight increase in CPD under dark conditions is attributed to the laser beam during the KPFM measurement. In contrast, the pristine perovskite thin film shows no significant CPD increase, indicating that continuous electron accumulation occurs only at the perovskite/C_60_ interface. After the light is turned off, the PSC level does not return to the initial state because of the band bending and trapped electrons formed by the reverse bias voltage. The repeated pulses will replicate and enhance this behavior, forcing the PSC to become larger. However, the remaining charges recombine with the removal of both incident light and bias voltage, causing the device current to instantly drop to its initial state. Compared to traditional von Neumann architecture-based computing systems, synaptic devices have the ability to perform data storage and processing with low energy consumption. For optoelectronic synapses, the energy consumption per event can be calculated using the following equation:

3 $$E=E_{ele}+E_{opt}=I\times V\times t+P\times A\times t$$

where *E*_ele_ is the electrical energy consumption, *E*_opt_ is the optical energy consumption, *V* is the applied bias voltage, *I* is the device current, *P* is the light intensity, *A* is the device area, and *t* is the duration of the optical pulse. Figure S12 presents a single synaptic event triggered by a 1 μW cm^–2^ light pulse of 0.2 s under a −0.1 V bias voltage. The energy consumption per event is calculated to be ∼67.6 pJ (electrical) and ∼10.2 nJ (optical), assuming an active area of 0.051 cm^2^. However, the energy consumption can be further reduced by decreasing the device’s active area. If the active area is reduced to 5.1 μm^2^, the energy consumption per synaptic event can be minimized to ∼10 fJ. The exceptionally low energy consumption, combined with the diverse synaptic functionalities, highlights the potential of the device for neuromorphic computing applications.

To demonstrate dual mode photodetection and synaptic behavior, a pattern contrast enhancement simulation was constructed. As shown in Figure S13, the photoresponse of the device with various bias voltages and incident light intensities was measured. At zero bias, the photoresponse of the device exhibits linear behavior with light intensity between 0 and 10 μW cm^–2^, indicating that the device operating in photodetector mode can faithfully render the indicated pattern. At reverse bias ranging from −1 to −3 V, the photoresponse shows significant variations with different light intensities compared to zero bias. As the light exposure time increases, the variation of the photoresponse also increases. The contrast enhancement simulation was performed by using the photoresponse measured from a single device, as shown in Figure 4(a). The photocurrent values used in the simulation were obtained by interpolating the experimental data shown in Figure S13 under the corresponding bias voltages and illumination conditions. Initially, an 8-bit grayscale pattern with low contrast was mapped to an incident light intensity range of 0 to 10 μW cm^–2^. The device array operating at zero bias was exposed to the mapped incident light. The resulting image, generated from the low-level photocurrent array under zero bias, reproduced the original contrast of the input pattern. Subsequently, the photocurrent array was fed back through an external circuit to generate a corresponding voltage array ranging from 0 to −3 V, which was then applied to the device array. Upon re-exposure to the same incident light, the devices operating under higher reverse bias exhibited enhanced photoresponse, thereby amplifying the contrast of the original pattern. Figure 4(b) presents the final simulation result. The original rabbit image with 40 × 40 pixels shows low contrast. After the feedback voltage is applied, the reconstructed image at 0 s shows increased contrast. With exposure times of 20, 40, and 60 s, the contrast is enhanced progressively, demonstrating the synaptic behavior of the device. To further verify the device behavior, an edge detection simulation was constructed (Figure 5(a)). During the convolutional process, the device array operates under a specific bias voltage pattern corresponding to a specific convolution kernel. As shown in Figure 5(b), horizontal and vertical edge detection kernels are applied to the device array. The simulation results are shown in Figure 5(c). After the convolution, the horizontal and vertical edges of the image are extracted from the original image, demonstrating the device’s capability for computing.

**Figure 4:**
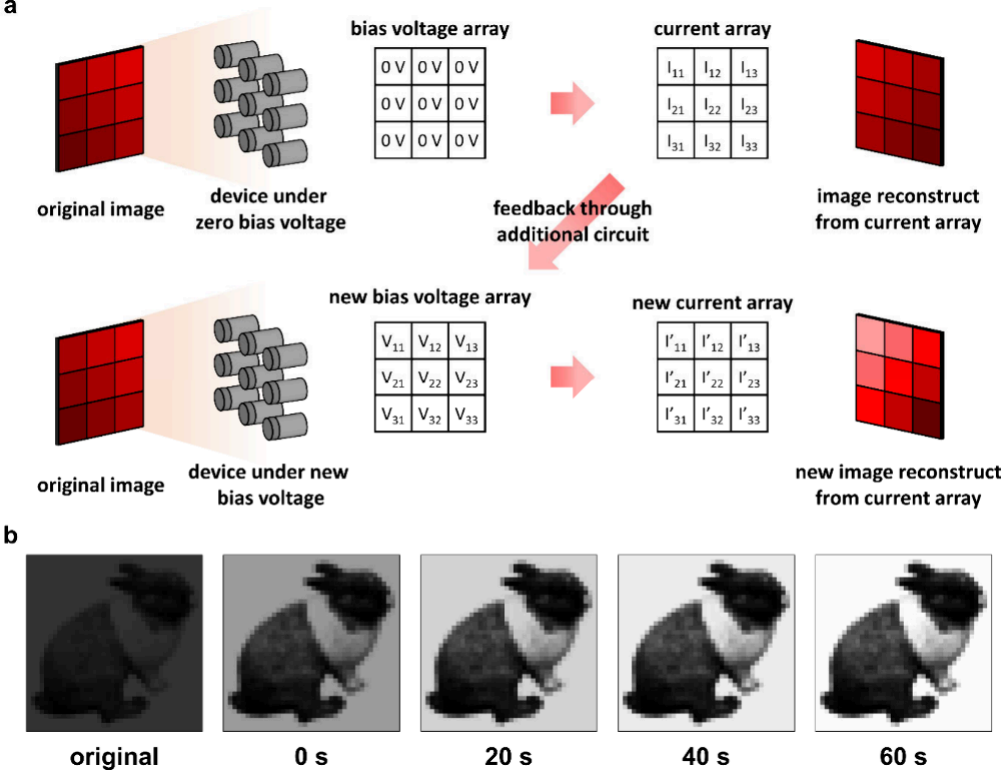
(a) Schematic representation of contrast enhancement with the devices. The different currents measured by the device array at zero bias are fed back to new reverse bias voltages array, leading to a larger difference in new current values when exposed to the pattern. (b) The original rabbit image consists of 40 × 40 pixels and the images reconstructed by the device array with the feedback voltage during various exposure time.

**Figure 5:**
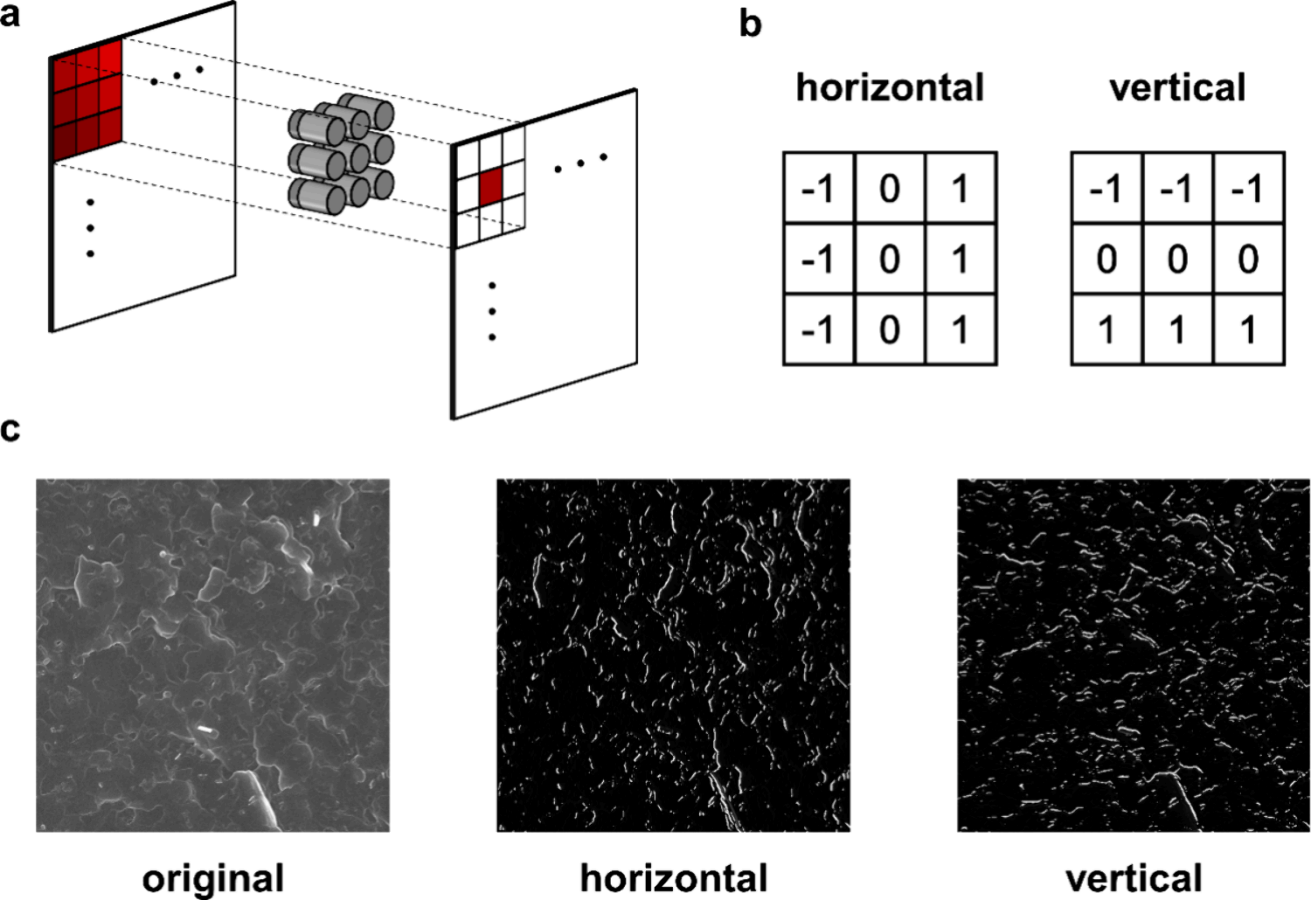
(a) Schematic representation of convolution with the devices for edge detection. (b) The 3 × 3 kernel of horizontal and vertical edge detection. (c) The original SEM image and the images after horizontal and vertical edge detection.

## Conclusion

In summary, a dual-mode artificial photonic synapse based on a lead-free PEA_2_SnI_4_ 2D Ruddlesden–Popper perovskite is demonstrated. As a photodetector, the device achieves a responsivity of 42.4 mA W^–1^, a high detectivity of 3.6 × 10^14^ Jones, and a broad LDR of 140 dB. In synaptic mode, the device successfully emulates synaptic behaviors, including PPF, LTM, and various forms of synaptic plasticity. Devices with different ETLs were compared, and synaptic behavior was achieved only with the incorporation of C_60_. Based on UPS data, the band alignment between PEA_2_SnI_4_ and C_60_ facilitates trapping of the photogenerated carriers at the interface, resulting in a larger PSC. Furthermore, the ability to enhance image contrast and perform edge detection was established, showcasing the device’s capability for photosensing and data memorization. These results highlight the potential of the dual-mode artificial photonic synapses for application in neuromorphic computing and artificial vision systems.

## Methods

### Materials

Phenethylammonium iodide (PEAI) was purchased from Greatcell Solar Materials. Tin iodide (SnI_2_, 99.99% trace metals basis), tin powder (99.8% trace metals basis), lithium fluoride (LiF, ≥99.99% trace metals basis), *N*,*N*-dimethylformamide (DMF, anhydrous, 99.8%), dimethyl sulfoxide (DMSO), and chlorobenzene (CB, anhydrous, 99.8%) were purchased from Sigma-Aldrich. Poly­(3,4-ethylenedioxythiophene):poly­(styrenesulfonate) (PEDOT:PSS) aqueous solution (Clevios PVP AI 4083) was purchased from Heraeus. Fullerene (C_60_, 99.9% purity) was purchased from Nichem. 2,4,6-Tris­[3-(diphenylphosphinyl)­phenyl]-1,3,5-triazine (PO-T2T, >99%) and 2,9-dimethyl-4,7-diphenyl-1,10-phenanthroline (BCP, >99%) was purchased from Lumtec.

### Device Fabrication

Patterned ITO glass substrates were cleaned by an ultrasonic cleaner with detergent, deionized (DI) water, acetone, and MeOH in sequence. Before usage, the substrates were cleaned with ultraviolet ozone for 10 min. The hole transport layer was fabricated by spin coating a filtered PEDOT:PSS aqueous solution on the substrates at 1000 rpm for 10 s followed by 5000 rpm for 30 s. The film was then annealed on a hot plate at 135 °C for 20 min. The PEA_2_SnI_4_ perovskite precursor solution was prepared in a N_2_-filled glovebox by mixing PEAI (398.5 mg, 1.6 mmol) and SnI_2_ (298 mg, 0.8 mmol) in a solvent mixture of 0.8 mL DMF and 0.2 mL DMSO to reach a concentration of 0.8 M. Sn powder (20 mg/mL) was added to prevent further oxidation of Sn^2+^. The precursor solution was stirred overnight and filtered before using. To fabricate the perovskite film, the precursor solution was spun onto the substrate at 4000 rpm for 65 s, and an antisolvent of CB was quickly dripped onto the surface at 5 s before the end of the spinning. The film was then annealed on a 100 °C hot plate for 20 min. The substrate was transferred into a high-vacuum chamber and pumped down to a pressure of 2 × 10^–6^ Torr. C_60_ (30 nm), LiF (0.5 nm), and Al (140 nm) were deposited sequentially onto the perovskite film by thermal evaporation, and all of the deposition rates were monitored by quartz crystal microbalance sensors.

### Device Characterization

The absorption spectra were acquired by using a Shimadzu UV-2600 UV–vis spectrophotometer. SEM images were obtained by using a JEOL JSM-IT800 scanning electron microscope. The XRDs were conducted using a Bruker D8 X-ray diffractometer with Cu Kα radiation. The GIWAXS measurements were conducted at beamline 23A1 (BL 23A1) at the National Synchrotron Radiation Research Center (NSRRC) in Taiwan. The data were performed by using a monochromatic X-ray beam with a wavelength of 1.2398 Å at an incident angle of 2.0°. The scatter signals were collected by a flat-panel detector (C10158DK with 2352 pixels), where the distance between the sample and the detector was 19.6 cm and the collection time was 5 s for each measurement. UPS information was acquired using ULVAC-PHI, PHI 5000 VersaProbe III. The *J*–*V* characteristics of the devices were measured using a Keysight B1500A semiconductor device parameter analyzer in a dark environment, and AM 1.5G solar illumination was obtained using a xenon lamp solar simulator. AFM and KPFM images were obtained using a scanning probe microscope (Bruker, model: Dimension ICON). The EQE spectrum and responsivity was acquired using the lock-in technique by using a current preamplifier (SR570, Stanford Research System) followed by a lock-in amplifier (SR860, Stanford Research System). The noise current of the device was calculated by conducting FFT of the dark currents measured by using a Keysight B1500A semiconductor device parameter analyzer. The transient photocurrent was measured using a 337 nm N_2_ laser (MNL330, LTB Lasertechnik Berlin) as the excitation source. A variable-gain high-speed current amplifier (DHPCA-100, FEMTO) was used to amplify the transient signals, which were recorded by using a Tektronix oscilloscope (DPO3050). The bandwidth of the device was determined by calculating the FFT of the transient photocurrent. The temporal responses and synaptic behaviors of the device were measured using a Keithley SourceMeter 2636B under the illumination of a 625 nm LED controlled by a LabVIEW program. The top Al electrode and the bottom ITO electrode were defined as the cathode and anode. Under reverse bias, a negative voltage was applied to the ITO electrode, while the Al electrode was kept at 0 V. According to this definition, a negative current flowing from the Al electrode to the ITO electrode was measured. The light intensity was calibrated using a NIST-traceable power meter (Ophir). The simulations of image contrast enhancement and edge detection were performed using homemade Python code with the photoresponse of a single device.

## Supplementary Material



## Notes

Absorption of PEA_2_SnI_4_ thin film annealed with different temperature; AFM image of the PEA_2_SnI_4_ perovskite thin film; the integrated intensity along the *qz* direction of GIWAX measurements of PEA_2_SnI_4_ thin film; noise current of the Keithley instrument and PEA_2_SnI_4_ device; double sweep *I*–*V* characteristics of the device; photoresponse of the PEA_2_SnI_4_ device under −3 V with the interval between light stimuli varied from 1 to 10 s; *I*–*t* curve of the PEA_2_SnI_4_ device under −3 V bias voltage without illumination; photoresponse of the device with different light pulse intensities; photoreponse of the devices with ETL of C_60_/PO-T2T, PO-T2T, C_60_/BCP, and BCP; UPS spectral evolution of various C_60_ thicknesses on PEA_2_SnI_4_ films; KPFM image of perovskite/C_60_ thin film under dark condition and white light illumination; photoresponse of the PEA_2_SnI_4_ device with the optical pulse of 1 μW cm^–2^ and the pulse width of 0.2 under −0.1 V bias voltage; photoresponse of the PEA_2_SnI_4_ device under various incident light intensities, bias voltages, and light exposure time; comparison of energy consumption and response speed in reported perovskite photonic synapses (PDF)
