# Supplementary material for: Dual-Mode Photonic Synapse Based on a Lead-Free 2D Ruddlesden–Popper Perovskite for Neuromorphic Vision
Source: ACS Appl Mater Interfaces. 2025 Aug 16;17(34):48547–54. doi: 10.1021/acsami.5c10557 (PMC12400261; doi:10.1021/acsami.5c10557)
Supplement: Supplementary file 1 [file am5c10557_si_001.pdf]

Support Information

Dual-Mode Photonic Synapse Based on a Lead-Free 2D Ruddlesden–Popper Perovskite for Neuromorphic Vision

Cheng-Yueh Chen[a], Hao-Cheng Lin[a], Pei-En Jan[a], Hung-Ming Chen[a], Yung-Tang Chuang[a], Chia-Feng Li[c], Yu-Ching Huang[c], and Hao-Wu Lin[a,b,]*

a. Department of Materials Science and Engineering, National Tsing Hua University, Hsinchu 30013, Taiwan.

b. Research Center for Critical Issues, Acdamia Sinica, Tainan 711, Taiwan.

c. Department of Materials Engineering, Ming Chi University of Technology, New Taipei City 24301, Taiwan

*To whom correspondence should be addressed.

E-mail: hwlin@mx.nthu.edu.tw (H. W. Lin)

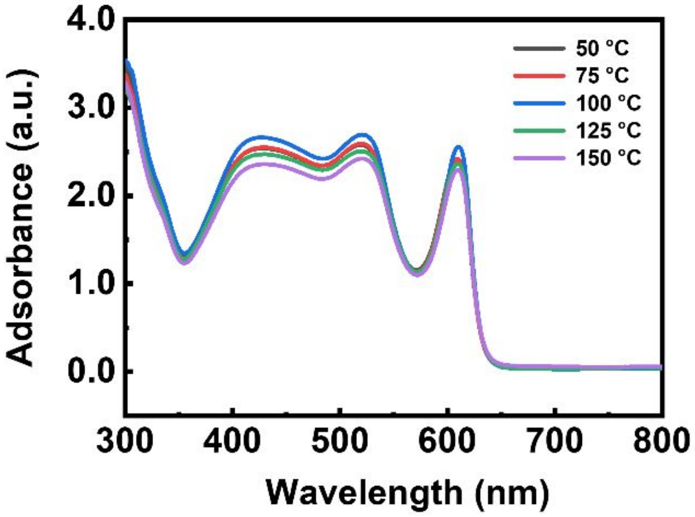

Figure S1. Absorption of PEA_2SnI_4 thin films annealed with different temperature ranging from 50 °C to 150 °C.

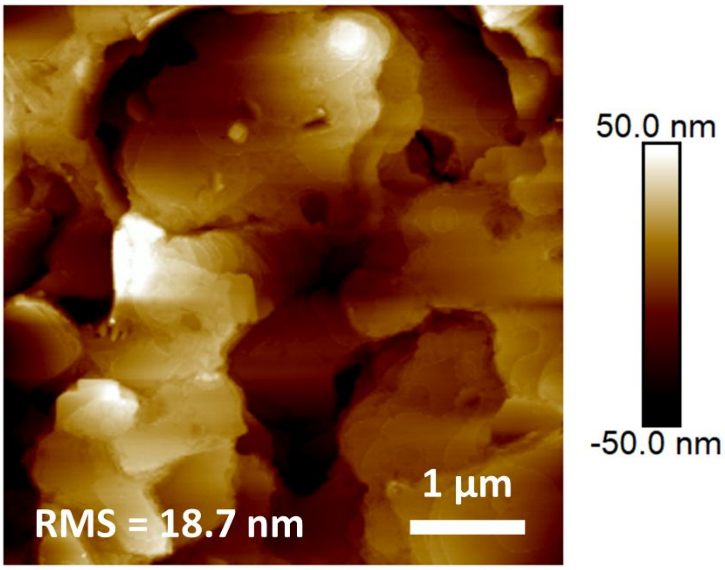

Figure S2. AFM image of the PEA_2SnI_4 perovskite thin film. The root mean square roughness of the thin film is calculated to be 18.7 nm.

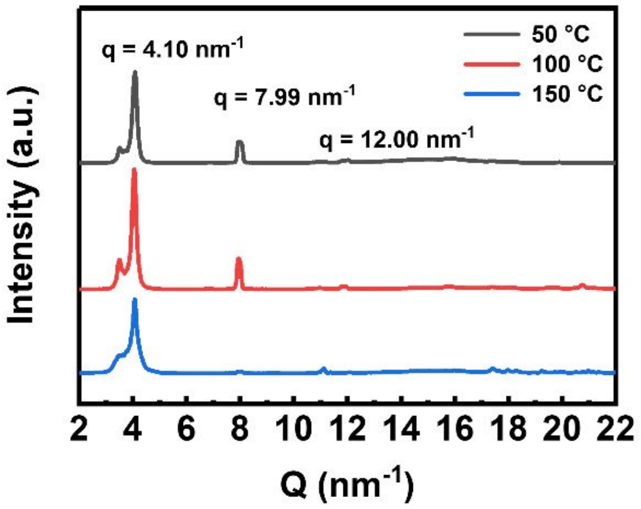

Figure S3. The integrated intensity along q_z direction from GIWAXS measurements of PEA_2SnI_4 films with different annealing temperatures.

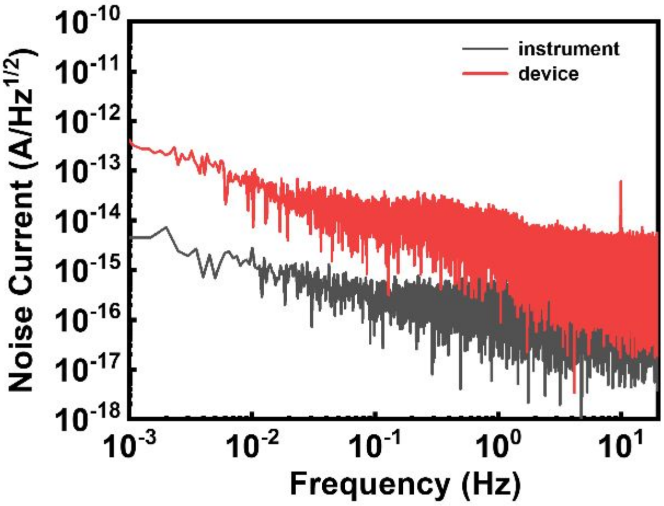

Figure S4. Noise current of the instrument and device obtained by the measured dark current.

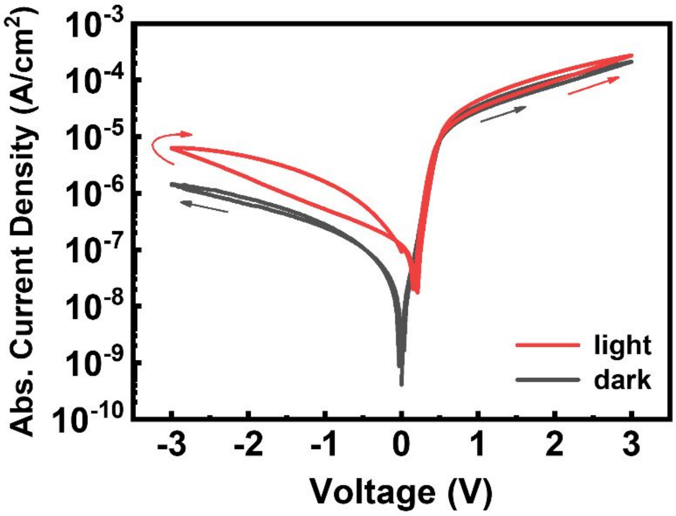

Figure S5. I-V characteristics of the device under a 625 nm LED illumination at 1 μW cm^{-2} (red line) and dark (black line) environment. Abs.: absolute.

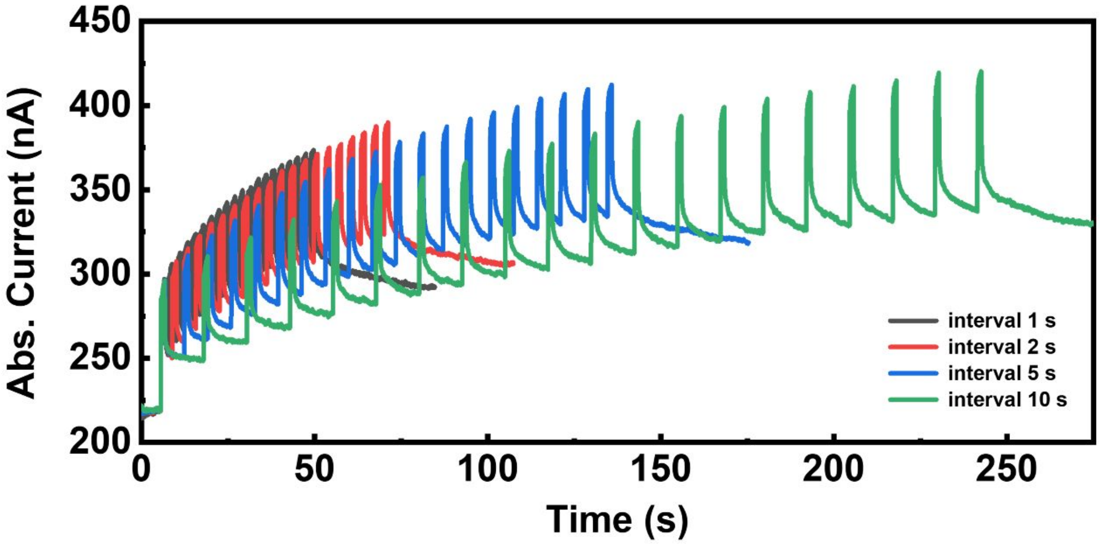

Figure S6. Photoresponse of the PEA_2SnI_4 device under -3V with the interval between light stimuli varied from 1 to 10 s. The width of each optical pulse is 1 s.

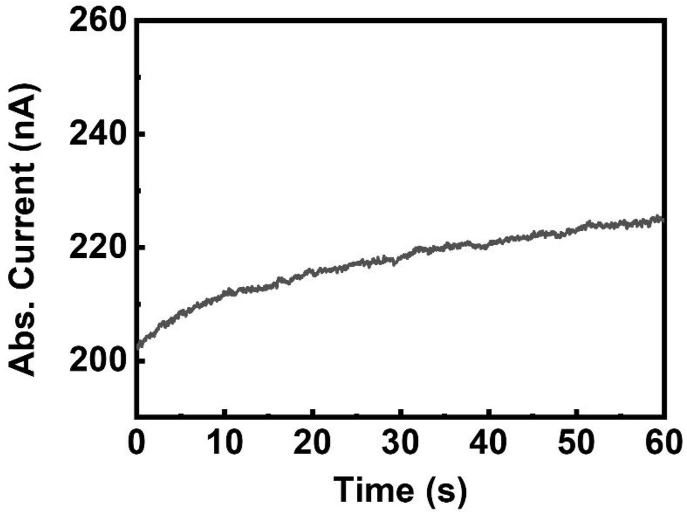

Figure S7. I-t curve of the PEA_2SnI_4 device under -3 V bias voltage without illumination.

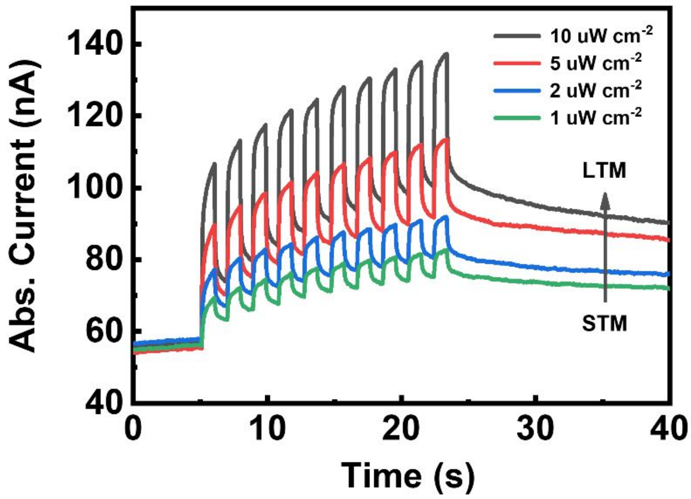

Figure S8. Photoresponse of the device with different light pulse intensities. The width and interval of each optical pulse are 1 s.

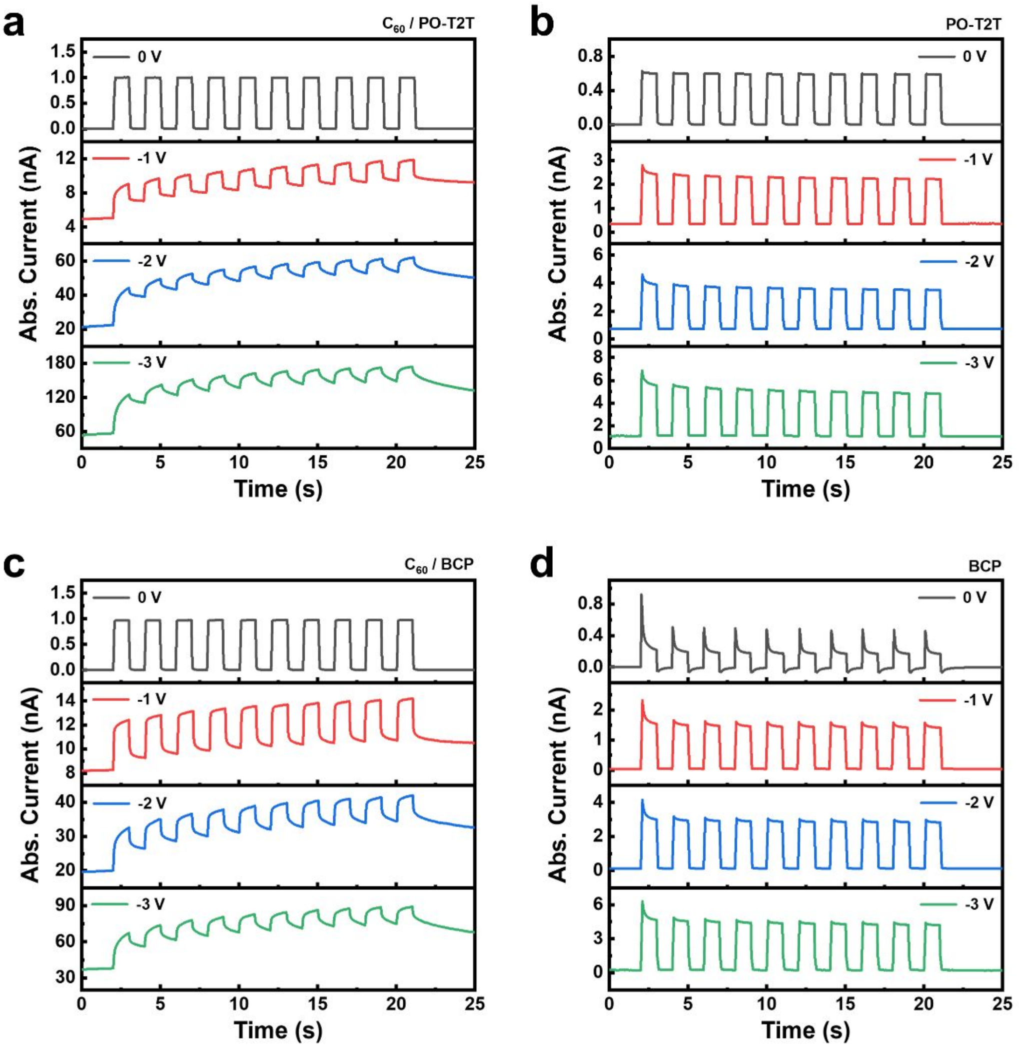

Figure S9. Photoresponse of the device under bias voltages ranging from 0 V to -3 V with ETL of (a) C_{60}/PO-T2T, (b) PO-T2T, (c) C_{60}/BCP, and (d) BCP.

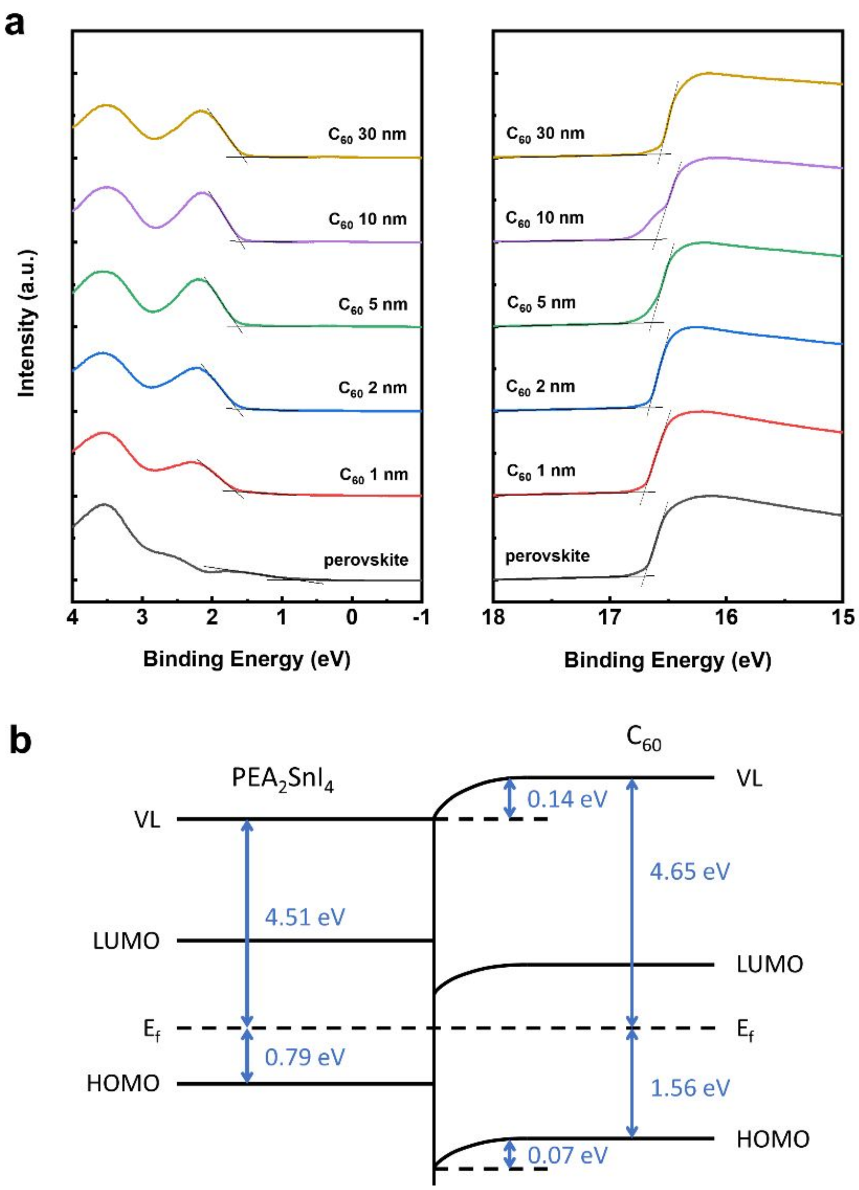

Figure S10. (a) UPS spectral evolution of various C_{60} thicknesses on PEA_2SnI_4 films. The left-hand onset of the highest occupied molecular orbital (HOMO) peak indicate position from the HOMO energy level to the Fermi level (E_f). The right-hand onset indicates the evolution of the work function (WF) from the vacuum level (VL) to the Fermi level (E_f). The work function can be calculate as WF = hν − E_{onset}, where hν is He I radiation of 21.2 eV, and E_{onset} is the photon binding energy corresponding to the secondary-electron onset. (b) The energy band diagrams of the device structure obtained from the above onset values.

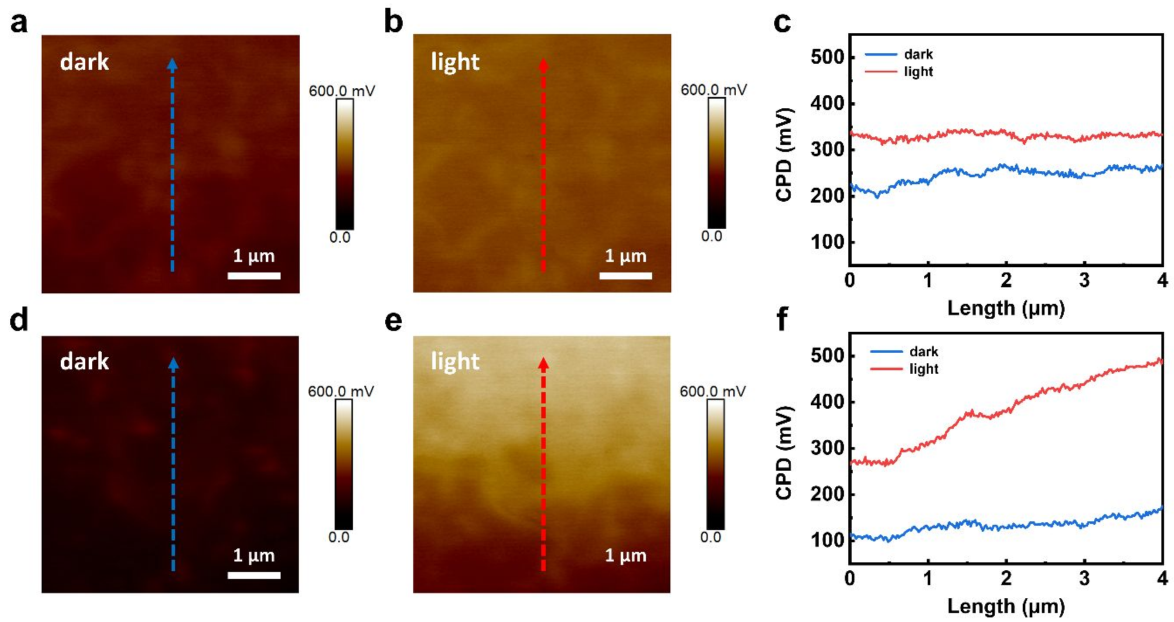

Figure S11. KPFM images of the pristine perovskite thin film under (a) dark conditions and (b) white-light LED illumination. (c) Contact potential difference (CPD) along the dashed lines in (a) and (b). KPFM images of the perovskite/C_{60} (1 nm) thin film under (d) dark conditions and (e) white-light LED illumination. (f) CPD along the dashed lines in (d) and (e).

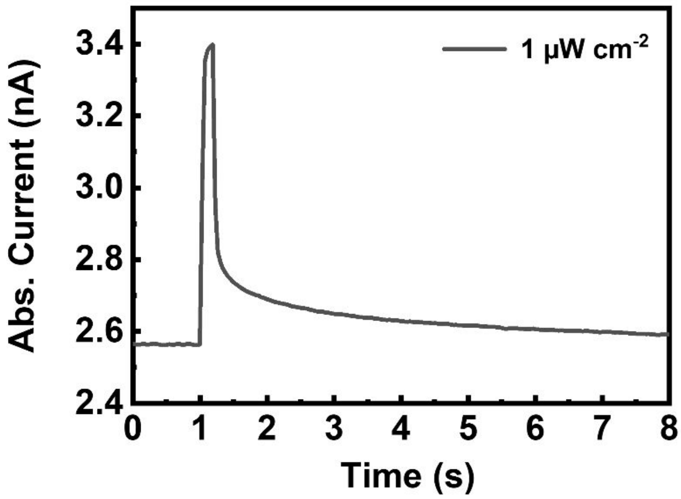

Figure S12. Photoresponse of the PEA_2SnI_4 device with the optical pulse of 1 μW cm^{-2} and the pulse width of 0.2 under -0.1 V bias voltage.

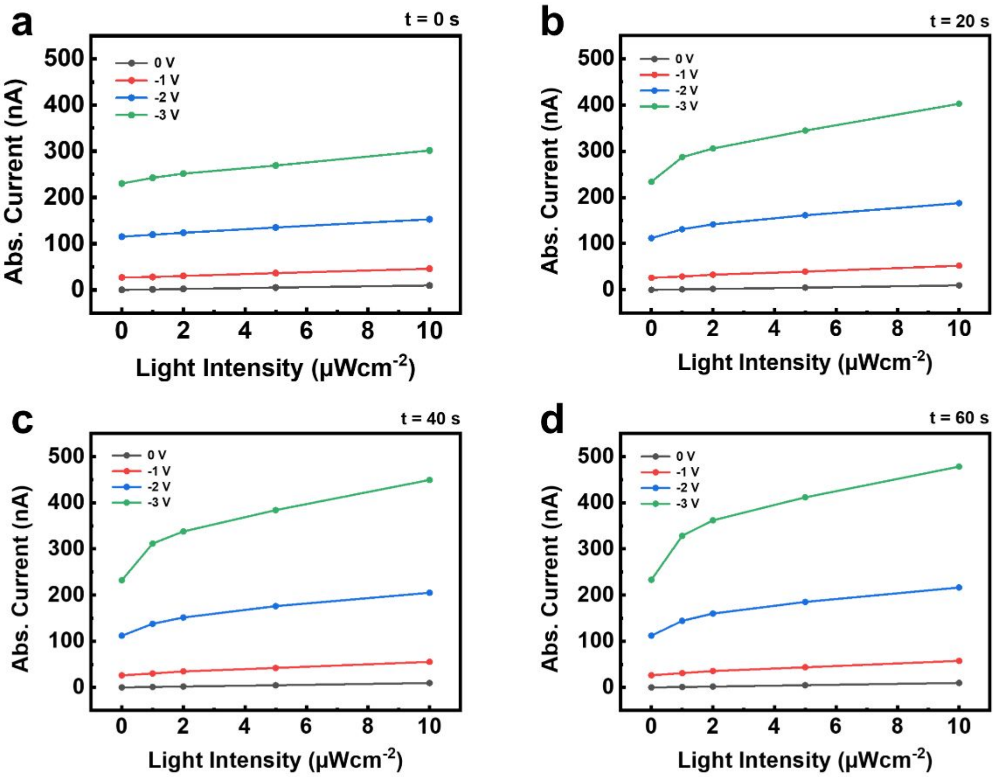

Figure S13. Photoresponse of the PEA_2SnI_4 device under various incident light intensities and bias voltages with light exposure times of (a) 0 s, (b) 20 s, (c) 40 s, and (d) 60 s.

Table S1. Comparison of energy consumption and response speed in reported perovskite photonic synapses.

type	Materials	Power Consumption	Response Speed	Ref.
3-terminal	$CsPbBr_3$/PDPP4T	325 μJ cm^{-2}	-	[1]
3-terminal	$CsPbBr_3$ QDs/PMMA/pentacene	41 μJ cm^{-2}	-	[2]
2-terminal	$(BA)_2PbI_4$	35 μJ cm^{-2}	6.86 s	[3]
3-terminal	DPPDTT + $CsPbBr_3$ QDs	2.5 μJ cm^{-2}	-	[4]
3-terminal	Si NM/$MAPbI_3$	200 nJ cm^{-2}	-	[5]
3-terminal	$CsPbBr_3$ QDs/P3HT	250 nJ	-	[6]
2-terminal	ZnO/PEA_2SnI_4	17 μJ cm^{-2}	-	[7]
2-terminal	$(4\text{-Cl-PEA})_2SnI_4$	12.32 μJ cm^{-2}	-	[8]
3-terminal	PEA_2SnI_4	232 nJ cm^{-2}	-	[9]
3-terminal	$FASnI_3$ + quercetin	185 nJ cm^{-2}	-	[10]
2-terminal	PEA_2SnI_4	200 nJ cm^{-2}	3.73 μs	This work

References

(S1) Chen, T.; Wang, X.; Hao, D.; Dai, S.; Ou, Q.; Zhang, J.; Huang, J. Photonic Synapses with Ultra-Low Energy Consumption Based on Vertical Organic Field-Effect Transistors. *Adv. Opt. Mater.* **2021**, *9*, 2002030.

(S2) Wang, Y.; Lv, Z.; Chen, J.; Wang, Z.; Zhou, Y.; Zhou, L.; Chen, X.; Han, S.-T. Photonic Synapses Based on Inorganic Perovskite Quantum Dots for Neuromorphic Computing. *Adv. Mater.* **2018**, *30*, 1802883.

(S3) Wang, Y.; Zha, Y.; Bao, C.; Hu, F.; Di, Y.; Liu, C.; Xing, F.; Xu, X.; Wen, X.; Gan, Z.; et al. Monolithic 2D Perovskites Enabled Artificial Photonic Synapses for Neuromorphic Vision Sensors. *Adv. Mater.* **2024**, *36*, 2311524.

(S4) Hao, D.; Zhang, J.; Dai, S.; Zhang, J.; Huang, J. Perovskite/Organic Semiconductor-Based Photonic Synaptic Transistor for Artificial Visual System. *ACS Appl. Mater. Interfaces* **2020**, *12*, 39487.

(S5) Yin, L.; Huang, W.; Xiao, R.; Peng, W.; Zhu, Y.; Zhang, Y.; Pi, X.; Yang, D. Optically Stimulated Synaptic Devices Based on the Hybrid Structure of Silicon Nanomembrane and Perovskite. *Nano Lett.* **2020**, *20*, 3378.

(S6) Gupta, G. K.; Kim, I.-J.; Park, Y.; Kim, M.-K.; Lee, J.-S. Inorganic Perovskite Quantum Dot-Mediated Photonic Multimodal Synapse. *ACS Appl. Mater. Interfaces* **2023**, *15*, 18055.

(S7) Yang, Y.; Li, Y.; Chen, D.; Shen, G. Multicolor vision perception of flexible optoelectronic synapse with high sensitivity for skin sunburn warning. *Mater. Horiz.* **2024**, *11*, 1934.

(S8) Liu, T.; Wang, H.; Sun, C.; Yuan, Z.; Wang, X.; Wang, L.; Wang, J.; Wang, S.; Zhang, Q.; Huang, L.; et al. Suppression of Tin Oxidation via Sn→B Bonding Interactions for High-Resolution Lead-Free Perovskite Neuromorphic Imaging Sensors. *Adv. Mater.* **2025**, 2502015.

(S9) Sun, Y.; Qian, L.; Xie, D.; Lin, Y.; Sun, M.; Li, W.; Ding, L.; Ren, T.; Palacios, T.

Photoelectric Synaptic Plasticity Realized by 2D Perovskite. *Adv. Funct. Mater.* **2019**, *29*, 1902538.

(S10) Liu, T.; Yuan, Z.; Wang, L.; Shan, C.; Zhang, Q.; Chen, H.; Wang, H.; Wu, W.; Huang, L.; Chai, Y.; et al. Chelated tin halide perovskite for near-infrared neuromorphic imaging array enabling object recognition and motion perception. *Nat. Commun.* **2025**, *16*, 4261.
